# The Zebrafish (*Danio rerio*) Is a Relevant Model for Studying Sex-Specific Effects of 17β-Estradiol in the Adult Heart

**DOI:** 10.3390/ijms20246287

**Published:** 2019-12-13

**Authors:** Selina Hein, David Hassel, Georgios Kararigas

**Affiliations:** 1 Department of Cardiology, Angiology and Pneumology, University Hospital Heidelberg, 69120 Heidelberg, Germany; 2 DZHK (German Centre for Cardiovascular Research), partner site Heidelberg/Mannheim, Germany; 3 Charité—Universitätsmedizin Berlin, Corporate Member of Freie Universität Berlin, Humboldt-Universität zu Berlin, Berlin Institute of Health, 10117 Berlin, Germany; 4 DZHK (German Centre for Cardiovascular Research), partner site Berlin, Germany

**Keywords:** cardiac, cardiovascular disease, contractility, estrogen, sex differences

## Abstract

Cardiovascular diseases are a major cause of morbidity and mortality, and there are significant sex differences therein. However, the underlying mechanisms are poorly understood. The steroid hormone 17β-estradiol (E2) is thought to play a major role in cardiovascular sex differences and to be protective, but this may not hold true for males. We aimed at assessing whether the zebrafish is an appropriate model for the study of E2 effects in the heart. We hypothesized that E2 regulates the cardiac contractility of adult zebrafish in a sex-specific manner. Male and female zebrafish were treated with vehicle (control) or E2 and the cardiac contractility was measured 0, 4, 7 and 14 days after treatment initiation using echocardiography. There was no significant effect on the heart rate by E2. Notably, there was a significant decrease in the ejection fraction of male zebrafish treated with E2 compared with controls. By contrast, there was no major difference in the ejection fraction between the two female groups. The dramatic effect in male zebrafish occurred as early as 4 days post treatment initiation. Although there was no significant difference in stroke volume and cardiac output between the two male groups, these were significantly higher in female zebrafish treated with E2 compared with controls. Gene expression analysis revealed that the levels of estrogen receptors were comparable among all groups. In conclusion, our data demonstrate that the adult zebrafish heart robustly responds to E2 and this occurs in a sex-specific manner. Given the benefits of using zebrafish as a model, new targets may be identified for the development of novel cardiovascular therapies for male and female patients. This would contribute towards the realization of personalized medicine.

## 1. Introduction

Cardiovascular diseases are the number one cause of death globally [1]. Notably, there are pronounced sex differences in the development, progression and outcome of cardiovascular diseases, as well as in the response to cardiovascular pharmacotherapies [2,3]. The steroid hormone 17β-estradiol (E2) is thought to be one of the major factors associated with age-specific cardiac remodeling [4], as well as for mediating sex-specific effects in the heart. Along this line, we previously reported direct effects of E2 in the mouse heart [5,6,7,8] and also E2-mediated cardiac effects that may differ significantly between the sexes [9,10,11,12,13]. Although E2 is believed to be cardioprotective in females, it may exert deleterious effects in males [10,14]. However, the underlying mechanisms and the contributing factors are incompletely understood. Consequently, further research is required employing appropriate and relevant animal models to unravel the actions of E2 in cardiac physiology.

The zebrafish (*Danio rerio*) has become an important model for elucidating disease mechanisms and has helped identify new genes and modifiers contributing to human heart muscle diseases [15,16]. In this context, we recently reported an isoproterenol-inducible heart failure model in adult zebrafish [17]. Interestingly, our previous work with zebrafish suggested major sex differences in cardiac function. We found that mutant zebrafish with lethal cardiac arrhythmias can be raised to adulthood under certain conditions [18]. However, predominantly female fish survived, clearly suggesting sex differences in the cardiac physiology of the zebrafish. However, the role of E2 in the heart of adult zebrafish, any of its effects on cardiac physiology, and its contribution to sex differences are not known.

The aim of this study was to assess whether the zebrafish is useful in investigating E2 effects in the heart. We tested the hypothesis that E2 regulates the cardiac contractile function of adult zebrafish in a sex-specific manner. In particular, based on our previous findings of E2 effects on cardiomyocyte contractile function [10], we further hypothesized that E2 treatment will lead to impaired cardiac contractility in male zebrafish.

## 2. Results

### 2.1. Study Design

**Figure 1:**
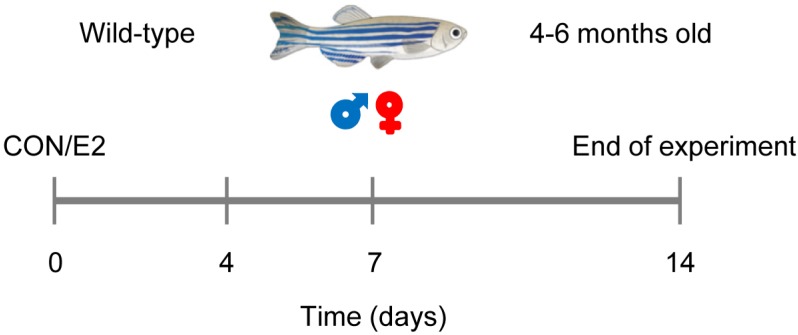
Study design. Male and female zebrafish (4–6 months old) were treated with vehicle (control, CON) or 17β-estradiol (E2, 0.1 μM). Cardiac function was assessed by echocardiography at 0, 4, 7 and 14 days after treatment initiation (*n* = 5/group).

In the present study, we investigated whether the zebrafish is a relevant model for the analysis of E2 effects in the adult heart under physiological conditions. We tested the hypothesis that E2 will affect the contractile function of the zebrafish heart in a sex-specific manner. For this purpose, we treated male and female zebrafish with vehicle (control, CON) or a dose of E2 (0.1 μM) that has been previously shown to exert estrogenic effects in zebrafish [19,20]. The contractile function of the heart was measured 0, 4, 7 and 14 days post treatment initiation using echocardiography (Figure 1).

### 2.2. E2 Effects on Cardiac Function

**Figure 2:**
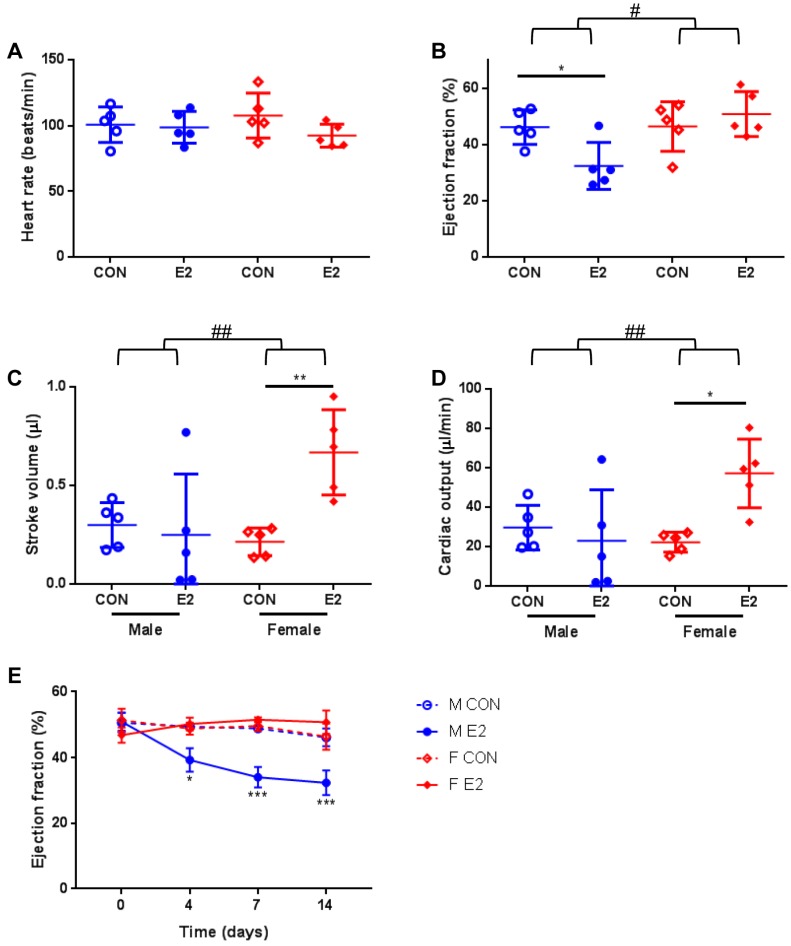
Echocardiographic analysis of male and female zebrafish treated with or without E2. (**A**) Heart rate, (**B**) ejection fraction, (**C**) stroke volume, and (**D**) cardiac output 14 days after treatment initiation. Data are presented in scatter dot plots including mean ± SD. (**E**) Ejection fraction assessed at 4, 7 and 14 days after treatment initiation. M: male; F: female; CON: control; E2: 17β-estradiol. Data present mean ± SEM; *n* = 5/group; # ANOVA interaction *p* ≤ 0.05; ## ANOVA interaction *p* ≤ 0.01; * *p* ≤ 0.05; ** *p* ≤ 0.01; *** *p* ≤ 0.001

All zebrafish were monitored throughout the study and there was no sign of toxicity. In our evaluation of how the adult zebrafish heart responds to a 14-day treatment with E2, the echocardiographic measurements revealed that there was no significant effect on the heart rate in any of the groups treated with E2 compared with the corresponding controls (Figure 2A). As hypothesized, the echocardiographic analysis demonstrated a marked impairment of systolic function in E2-treated male zebrafish only (ANOVA interaction *p* = 0.019 and F = 6.691). In particular, there was a significant decrease in the ejection fraction of male zebrafish treated with E2 compared with controls (adjusted *p* ≤ 0.05) (Figure 2B). In contrast, there was no major difference in the ejection fraction between the E2-treated female zebrafish and controls (Figure 2B). Interestingly, there was no significant difference in stroke volume and cardiac output between the two male groups (Figure 2C,D). However, both stroke volume (ANOVA interaction *p* = 0.012 and F = 7.901) and cardiac output (ANOVA interaction *p* = 0.014 and F = 7.651) were significantly increased in female zebrafish treated with E2 compared with controls [adjusted *p* ≤ 0.01 (stroke volume), adjusted *p* ≤ 0.05 (cardiac output)] (Figure 2C,D).

On the basis of the longitudinal data available, we sought to identify the earliest time point that E2 exerts this dramatic effect in male zebrafish. We found that male zebrafish treated with E2 already at day 4 exhibited a significant systolic dysfunction associated with impaired cardiac contractility as measured by ejection fraction (Figure 2E). By contrast, there was no significant effect at any time point in female zebrafish treated with E2 compared with controls (Figure 2E). Collectively, our data demonstrate that the adult zebrafish heart robustly responds to E2 and that this occurs in a sex-specific manner.

### 2.3. Assessment of Estrogen Receptor Levels

**Figure 3:**
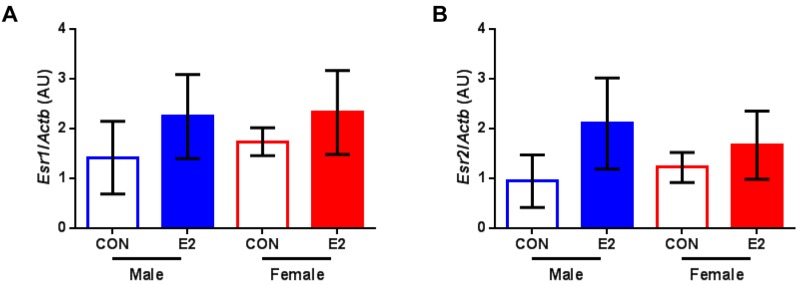
Gene expression analysis of estrogen receptors. (**A**) Relative mRNA levels of estrogen receptor alpha (*Esr1*) and (**B**) estrogen receptor beta (*Esr2*) in hearts of male and female zebrafish treated with vehicle (control, CON) or 17β-estradiol (E2) normalized to beta actin (*Actb*). Data present mean ± SD; *n* = 3/group; each replicate was derived from 2–5 pooled hearts; AU: arbitrary unit.

Given the sex-specific effects of E2 in the heart of adult zebrafish, we then asked whether there are any effects on the gene expression levels of estrogen receptors. Our analysis revealed that the levels of both estrogen receptor alpha (Figure 3A) and estrogen receptor beta (Figure 3B) were comparable among all groups.

## 3. Discussion

The present findings show that the zebrafish is an appropriate model for the study of E2 effects in the adult heart. Of note, we found that E2 regulates the cardiac contractile function of zebrafish in a sex-specific manner. In particular, male zebrafish treated with E2 exhibited a significant decrease in ejection fraction compared with controls, while ejection fraction was not affected in female zebrafish.

In men, E2 levels are markedly increased with obesity [21] and advancing age [22,23], and elderly men may have higher concentrations of E2 compared with age-matched women [24]. Previous studies have implicated elevated E2 levels with increased risk of cardiovascular disease in men. In particular, elevated E2 levels in men are associated with coronary artery disease and myocardial infarction [25] and with an increased risk of stroke [22]. Of note, abnormally high E2 concentrations are a significant predictor of poor prognosis and higher mortality in men with chronic heart failure and reduced left ventricular ejection fraction [26]. However, the underlying mechanisms are poorly understood.

Consequently, appropriate and relevant animal models are necessary for the elucidation of underlying molecular mechanisms and, in turn, the drug development process. Given that the zebrafish genome has been sequenced and annotated, and that most zebrafish genes are highly conserved in mammals with a zebrafish ortholog identified for ∼70% of human genes [27], the zebrafish has proven to be a valuable in vivo model for identifying underlying mechanisms and to evaluate novel disease genes [28,29]. Along this line, the zebrafish has become an important tool for high-throughput therapeutic molecule testing, as compounds can be simply added to the water, and for the identification of novel targets with therapeutic potential [30,31]. However, the role of E2 in the heart of zebrafish and any potential effects on cardiac physiology are not known.

In the present study, we found that after exposure to E2 for 14 days, the ejection fraction markedly declined in male zebrafish, indicating cardiac dysfunction. Notably, the male-specific decrease in systolic function mediated by E2 was already evident at 4 days post treatment initiation (earliest time point of available echocardiographic data). By contrast, there was no major effect on the ejection fraction of female zebrafish at any time point. Interestingly, the female zebrafish demonstrated an increase in stroke volume and cardiac output in response to E2. In a previous study of E2 effects on cardiomyocytes isolated from mouse hearts, we noticed a tendency for higher contractility as assessed by cell shortening measurements in cardiomyocytes of female mice treated with E2 compared with controls [10]. Given that stroke volume depends on contractility among other factors, this could explain its observed increase in female zebrafish. As the underlying mechanisms of the sex-specific E2 effects in the heart are poorly understood, the zebrafish will be a useful tool in addressing this knowledge gap. Markedly, our gene expression analysis revealed that the levels of estrogen receptors were comparable among all groups. Further research is warranted.

## 4. Materials and Methods

### 4.1. Zebrafish Care

Zebrafish breeding and maintenance experiments on 4–6 months old wild-type male and female fish were performed as described previously [32]. All experimental protocols were approved by the institutional review board of the University of Heidelberg and the responsible government authority of Baden-Wuerttemberg, Germany (Nr. 35–9185.81/G-62 in 2015).

### 4.2. Treatment

As described previously [19,20], E2 solutions were added to the water of the zebrafish at the final concentration of 0.1 μM. To ensure a constant E2 concentration, the water, containing the respective amount of E2, was changed daily. The control treatment was 2-hydroxypropyl-β-cyclodextrin (vehicle, control for E2 treatment). Therefore, there were four groups, i.e., M CON (male control), F CON (female control), M E2 (male E2), and F E2 (female E2), with *n* = 5/group.

### 4.3. Echocardiography

Cardiac geometry and function were assessed by echocardiography as previously described [32,33]. Briefly, the Vevo2100 Imaging system (Visual Sonics, Amsterdam, The Netherlands) equipped with a high-frequency transducer (MS700, 30–70 MHz) was used to examine adult zebrafish sedated with 2-phenoxyethanol (3.2 μM in the maintenance water; Sigma-Aldrich, München, Germany). Two cardiac examination planes were recorded: a long axis view and an abdominal-cranial axis to attain pulsed wave Doppler measurements. We calculated ejection fraction and stroke volume by using the long axis view. We analyzed the heart rate applying the abdominal-cranial axis.

### 4.4. Quantitative RT-PCR

Reverse transcription and quantitative real-time-PCR were performed as described previously [34,35]. Total RNA was isolated using the RNeasy Mini Kit (Qiagen, Hilden, Germany) and reverse transcribed using the RevertAid H Minus cDNA Synthesis Kit (ThermoFisher Scientific, Darmstadt, Germany). Reactions where RNA or reverse transcriptase had been omitted were used as negative controls. PCR products were obtained using gene-specific primers reported previously [36] and SYBR Green (Applied Biosystems, Darmstadt, Germany) in a 7000 ABI Prism Instrument (Applied Biosystems, Darmstadt, Germany). The levels of the target genes were normalized to beta actin (*Actb*) housekeeping mRNA levels. The primer sequences were the following: *Esr1* forward 5’-CAGGACCAGCCCGATTCC-3’ and reverse 5’-TTAGGGTACATGGGTGAGAGTTTG-3’; *Esr2* forward 5’-CGCTCGGCATGGACAAC-3’ and reverse 5’-CCCATGCGGTGGAGAGTAAT-3’; *Actb* forward 5’-TGCCCCTCGTGCTGTTTT-3’ and reverse 5’-TCTGTCCCATGCCAACCAT-3’.

### 4.5. Statistical Analysis

Data are presented as mean ± SD. Statistical significance was assessed using the R version 2.14.2 software (The R Foundation for Statistical Computing, Vienna, Austria). Normality of the data was tested with the Shapiro-Wilk test. Comparisons among multiple groups were made with two-way ANOVA and Tukey’s post hoc test adjusting for multiple comparisons. *p* ≤ 0.05 was considered significant.

## 5. Conclusions

Several clinical and experimental studies demonstrate the implication of E2 in cardiovascular diseases. The molecular mechanisms responsible for sex-specific E2 effects in the heart and the association of circulating E2 levels with cardiac function and cardiovascular disease prognosis are poorly understood. A better understanding of the role of E2 in cardiac pathophysiology may help us to delineate future studies that would guide improvements in the knowledge and treatment of cardiovascular disease. On this point, this brief report shows that the zebrafish is a relevant model for the investigation of regulatory effects of E2 in the heart. Given the rapid maturation of zebrafish and that large-scale drug screens exploit the ease of treating zebrafish with drugs, this, in turn, may contribute to the rapid identification and confirmation of new targets for the development of novel therapies for clinical use in men and women. This would represent a crucial step towards the realization of personalized medicine and translation into clinical routine.

## Abbreviations

E2	17β-estradiol
